# A Clinical Roadmap to Investigate the Genetic Basis of Pediatric Pheochromocytoma: Which Genes Should Physicians Think About?

**DOI:** 10.1155/2018/8470642

**Published:** 2018-03-20

**Authors:** Bernardo Dias Pereira, Tiago Nunes da Silva, Ana Teresa Bernardo, Rui César, Henrique Vara Luiz, Karel Pacak, Luísa Mota-Vieira

**Affiliations:** ^1^Serviço de Endocrinologia e Nutrição, Hospital do Divino Espírito Santo de Ponta Delgada (EPER), Av. D. Manuel I, 9500-370 Ponta Delgada, Açores, Portugal; ^2^Serviço de Endocrinologia e Diabetes, Hospital Garcia de Orta (EPE), Av. Torrado da Silva, 2851-951 Almada, Setúbal, Portugal; ^3^Serviço de Cirurgia Geral, Hospital do Divino Espírito Santo de Ponta Delgada (EPER), Av. D. Manuel I, 9500-370 Ponta Delgada, Açores, Portugal; ^4^Section on Medical Neuroendocrinology, Eunice Kennedy Shriver NICHD, NIH, Building 10 CRC 1E-3140 10 Center Drive MSC-1109, Bethesda, MD 20892-1109, USA; ^5^Unidade de Genética e Patologia Moleculares, Hospital do Divino Espírito Santo de Ponta Delgada (EPER), Av. D. Manuel I, 9500-370 Ponta Delgada, Açores, Portugal; ^6^Biosystems & Integrative Sciences Institute (BioISI), Faculty of Sciences, University of Lisbon, Lisbon, Portugal; ^7^Instituto Gulbenkian de Ciência, Oeiras, Portugal

## Abstract

Pheochromocytoma is very rare at a pediatric age, and when it is present, the probability of a causative genetic mutation is high. Due to high costs of genetic surveys and an increasing number of genes associated with pheochromocytoma, a sequential genetic analysis driven by clinical and biochemical phenotypes is advised. The published literature regarding the genetic landscape of pediatric pheochromocytoma is scarce, which may hinder the establishment of genotype-phenotype correlations and the selection of appropriate genetic testing at this population. In the present review, we focus on the clinical phenotypes of pediatric patients with pheochromocytoma in an attempt to contribute to an optimized genetic testing in this clinical context. We describe epidemiological data on the prevalence of pheochromocytoma susceptibility genes, including new genes that are expanding the genetic etiology of this neuroendocrine tumor in pediatric patients. The clinical phenotypes associated with a higher pretest probability for hereditary pheochromocytoma are presented, focusing on differences between pediatric and adult patients. We also describe new syndromes, as well as rates of malignancy and multifocal disease associated with these syndromes and pheochromocytoma susceptibility genes published more recently. Finally, we discuss new tools for genetic screening of patients with pheochromocytoma, with an emphasis on its applicability in a pediatric population.

## 1. Introduction

Pheochromocytoma (PHEO; MIM #171300) is a rare neuroendocrine tumor of chromaffin cells originating in the adrenal medulla, whereas paragangliomas (PGLs; MIM #168000) are even rarer tumors arising in the paraganglia along the parasympathetic and sympathetic chains [1]. PHEO is rare among a pediatric population, representing 9.6–17.7% of all cases [2, 3]. In the last fifteen years, several new genes have been implicated in the development of this tumor [4, 5]. Besides the three classical PHEO-associated cancer syndromes, namely, multiple endocrine neoplasia type 2 (MEN2), von Hippel-Lindau (VHL) disease, and neurofibromatosis type 1 (NF1), new entities have been associated with PHEO: the PGL syndrome types 1 to 5 [(PGL1–5) caused by mutations in succinate dehydrogenase (SDH) subunits D/AF2/C/B/A genes (*SDHx*), resp.], familial PHEO [caused by mutations in Myc-associated protein X (*MAX*) and transmembrane protein 127 (*TMEM127*) genes] [6–15], and several new susceptibility genes. These include hypoxia-inducible factor 2 alpha (*HIF2A*), fumarate hydratase (*FH*), prolyl hydroxylase types 1 and 2 (*PHD1* and *PHD2*), Harvey rat sarcoma viral oncogene homolog (*HRAS*), kinesin family member 1B (*KIF1B*), and X-linked alpha thalassemia mental retardation (*ATRX*) genes [16–20]. Thus, nearly one-third of all patients with PHEO have germline mutations [5, 21], and this number is significantly higher at younger ages [3, 22]. Consequently, all patients ≤ 18 years old (yo) diagnosed with this tumor should be considered for genetic testing [23, 24]. Besides young age, there are other clinical features where a PHEO is more likely to be associated with a genetic etiology. Syndromic presentations, multifocal disease (adrenal and extraadrenal), bilateral and recurrent PHEO, and metastatic disease are all associated with a higher likelihood of a hereditary PHEO [3, 22].

Considering the growing number of PHEO susceptibility genes and the well-established genotype-phenotype correlations for some of these gene mutations [3, 22], it is recommended that clinicians follow an algorithm based on the phenotype when requesting a genetic analysis [22, 23]. Also, it may be clinically unsuitable to order a genetic study for genes that have never been associated with specific features of a patient's phenotype [23, 24]. Furthermore, two other reasons should be taken into account while considering genetic testing: (1) the high rate of metastatic PHEO associated with some mutations, which may aid in tailoring the appropriate follow-up [25], and (2) the finding of a mutation in the index case and their relatives, which allows for an individualized surveillance program to timely detect and treat chromaffin and other nonchromaffin cell tumors or disorders [26]. Additionally, when a genetic mutation is suspected to be linked to PHEO, other parameters may aid in the selection of the molecular analysis, namely, the type of catecholamine production [27] and/or the pattern of SDHA/B immunostaining in pathology specimens [28, 29] and a specific imaging phenotype [30].

Due to the rarity of pediatric PHEO, few cohort studies in this age range have been published that allow for precise genotype-phenotype correlations for all associated mutations. The majority are based on small samples and takes into account small susceptibility gene panels [2, 25, 31–37]. Thus, the amount of published data may hinder, at least in some cases, the appropriate selection of genetic analysis for a pediatric PHEO. Here, we review the clinical phenotypes of pediatric patients with PHEO and associated mutations in susceptibility genes reported in the literature, in an attempt to contribute to a comprehensive genetic screening of PHEO in pediatric age.

The present review was conducted taking into account the English literature retrieved from PubMed until August 2017. Main keywords used were “pheochromocytoma,” “paraganglioma,” “genetic testing,” “mutation,” “genotype,” “phenotype,” “malignant,” “metastatic,” “pediatrics,” “child,” “preschool child,” “VHL,” “RET,” “NF1,” “SDHB,” “SDHD,” “SDHA,” “SDHC,” “SDHAF2,” “TMEM127,” “MAX,” “HIF2A,” “FH,” “PHD1,” “PHD2,” “MDH2,” and “KIF1B.” We included clinical and molecular studies (single cases and case series); we did not exclude any age to minimize missing pediatric cases of cohort studies that considered all ages. We also considered leading reviews in the field of genetics of PHEO. We then excluded genes not reported to be associated with pediatric PHEO.

## 2. Mechanisms of Hereditary PHEO

The susceptibility genes involved in the development of pediatric PHEO may be grouped according to three primary mechanisms of oncogenesis: a pseudohypoxic cluster [mutations in *VHL*, *HIF2A* (or *EPAS1*), *PHD1*, *PHD2*, *FH*, *SDHx*, and *MDH2*], a cluster composed of kinase receptor signaling and protein translation pathways (mutations in *RET*, *NF1*, and *MAX*), and a Wnt-altered pathway cluster [38–40]. This last cluster comprises only somatic driver mutations (*CSDE1* truncating mutations, and *MAML3* fusion genes) that cause sporadic aggressive/recurrent PHEO [40].

In the pseudohypoxic cluster, there is a common denominator of overexpression of hypoxia-inducible factor alpha (HIF-*α*), which is expressed predominantly by its HIF-2*α* isoform in the neural crest cells [41, 42]. Under hypoxia (or pseudohypoxia, a condition where there is normal concentration of oxygen that is not consumed due to a defect in the oxygen sensor pathways), the cell develops a set of adaptive responses in which HIF-*α* plays a central role in regulating genes involved in erythropoiesis (e.g., *EPO*), angiogenesis (e.g., *ADM* and *VEGFA*), glucose metabolism (e.g., *HK1* and *HK2*), cellular proliferation (e.g., *TGFB* and *CCND1*), and survival (e.g., *BNIP3*). However, a long-standing process of hypoxia (or pseudohypoxia) causes HIF-*α* excess, which promotes a nuclear overexpression of these genes, ultimately leading to cancer development, migration, invasion, and metastasis [41, 42].

In the Krebs cycle, the SDH complex (formed by its catalytic subunits A and B, and anchorage subunits C and D) and FH enzymes catalyze the oxidation of succinate to fumarate and the conversion of fumarate to malate, respectively. Loss-of-function mutations in *SDHx* or *FH* lead to succinate and fumarate accumulation, respectively, and to a subsequent inhibition of HIF-*α* hydroxylation, a necessary signal recognition step for its degradation by the VHL protein [42, 43]. The PHD1 and PHD2 hydroxylate HIF-*α* isoforms and loss-of-function mutations in their genes (*PHD1* and *PHD2*) cause an excess of HIF-*α* and its proneoplastic actions [17, 42]. Mutations in *HIF-α* promote electrostatic changes in the protein isoforms, which impair hydroxylation by PHD molecules, preventing the signaling for degradation by the VHL protein [44, 45]. Finally, *VHL* mutations originate defective proteins that do not recognize hydroxylated HIF-*α* isoforms for degradation. This excess of HIF-*α* leads to an overexpression of hypoxia-related genes, favoring metastasis [44, 45].

The proto-oncogene *RET* encodes a transmembrane receptor tyrosine kinase involved in organ development (e.g., gut, kidney, and neural crest), proliferation, and apoptosis. Germline mutations in specific exons of the *RET* may lead to constitutive activation of its protein tyrosine kinase domain and subsequent downstream activation of Ras/mitogen-activated protein kinase and PI3 kinase/AKT pathways, promoting tumorigenesis through cell proliferation and reduced apoptosis [46]. Contrary to *RET*, the *NF1* acts as a tumor suppressor gene. Its protein—neurofibromin 1—is a GTPase activator that inhibits Ras signaling through the mTOR kinase pathway. Loss-of-function mutations in *NF1* result in an enhanced cell proliferation through impaired Ras signaling inhibition [47]. The *MAX* gene encodes a protein that acts as a nuclear transcriptional repressor of Myc. Loss-of-function mutations in *MAX* generate a protein incapable of binding to Myc in the nucleus, leading to proliferation, angiogenesis, and repression of cell differentiation [14, 48].

## 3. Genetic Basis of Pediatric PHEO: Which Genes Should We Think About?

### 3.1. Genetic Prevalence and PHEO-Associated Genes

Considering the largest cohorts of pediatric PHEO [31–33], where a wider set of genes were studied, the prevalence of gene mutations was estimated to be 68.2–80.0% in a total of 268 patients. To our knowledge, 10 genes have been described in association with PHEO at a pediatric age: *VHL* (MIM ∗608537), rearranged during transfection (*RET*; MIM +164761), *NF1* (MIM ∗613113), *SDHD* (MIM ∗602690), *SDHB* (MIM ∗185470), *SDHA* (MIM ∗600857), *MAX* (MIM ∗154950), *HIF2A* (MIM ∗603349), *FH* (MIM ∗136850), and *PHD1* (MIM ∗606424) [17, 24, 31–33, 49–51]. The other seven genes that have been associated with PHEO were only reported in adults. These genes are *TMEM127* (MIM ∗613403), *PHD2* (MIM ∗606425), *SDHAF2* (MIM ∗613019), *SDHC* (MIM ∗602413), *HRAS* (MIM ∗190020), *KIF1B* (MIM ∗605995), and *ATRX* (MIM ∗300032) [10, 11, 15, 17–20, 31–33, 52, 53].

### 3.2. PHEO-Associated Cancer Syndromes

A pediatric PHEO can be included in one of the following five cancer syndromes: VHL, MEN2, NF1, and those associated with *PHD1/2* and *HIF2A* mutations. The VHL disease (MIM #193300) is a highly penetrant, autosomal dominant syndrome characterized by central nervous system and retinal hemangiomas (60.0%), renal cysts (50.0–70.0%), renal cell carcinomas (RCC, 28.0%), PHEO (7.0–20.0%), and pancreatic neuroendocrine tumors (5.0–10.0%) and cysts. The VHL disease has an average age onset of 27 years, and by 65 yo, almost all carriers have developed clinical disease [7, 54, 55]. The most common cause of death is RCC, but this tumor almost always develops after 20 yo [7]. Pediatric PHEO tends to present earlier when *VHL* is mutated, compared with other PHEO-associated germline mutations: The mean age of diagnosis of PHEO is 12 years, and the youngest age reported to date is 4 years [31]. Large deletions and truncating mutations of *VHL* predispose to hemangiomas of central nervous system (including retina) and RCC, but not to PHEO (VHL disease type 1). Missense mutations predispose to PHEO (VHL disease type 2), which may be associated with hemangioblastomas (VHL disease type 2A), hemangioblastomas and RCC (VHL disease type 2B), or only PHEO (VHL disease type 2C) [56]. *VHL* mutations are the most prevalent in pediatric patients with PHEO, ranging from 28.0% to 49.0% of cases [31–33]. However, although syndromic features that raise suspicion for VHL disease have a high penetrance across the age spectrum [7, 56], pediatric patients with PHEO-associated *VHL* mutations often present without other syndromic features of the disease [31–33]. In the largest cohort of pediatric PHEO, published to date, only 10 of 93 VHL patients had the prototypic lesions at study entry [31]. Additionally, more than half of patients with VHL disease have de novo mutations; that is, the family history is unremarkable in these cases. Thus, in a seemingly sporadic case, clinicians should always bear in mind the relatively high frequency of *VHL*-germline mutations in pediatric PHEO [6, 31–33].

MEN2 is a syndrome caused by *RET* mutations inherited in an autosomal dominant pattern. It may be subdivided into two clinical subtypes: MEN2A (MIM #171400) and MEN2B (MIM #162300). MEN2A is the most prevalent, with a mean age of onset of disease (first prototypic tumor) of 37.5 years. Patients are susceptible to medullary thyroid carcinoma (MTC, 97.0%), PHEO (68.1%), and primary hyperparathyroidism (13.4%) [57]. MEN2A patients with specific mutations in *RET* codons 631 and 634 have the highest incidence of PHEO [58]. MEN2B patients are usually diagnosed earlier (average age of first prototypic tumor: 13–22 years) than are MEN2A individuals. MEN2B is associated with an aggressive form of MTC (100.0%; usually incurable if diagnosed ≥13 yo), PHEO (58.0%), marfanoid habitus, and ganglioneuromatosis of the gut and oral mucosa [57, 59–61]. Pediatric PHEO is part of MEN2 syndromes in 1.0–5.4% of cases [31–33]. Contrary to adults, where PHEO can be the first manifestation of MEN2 in 24.0–37.3% of cases [6, 57, 62], the vast majority of pediatric patients with MEN2 have a previous history of MTC or family history of typical tumors of the syndrome [62–64]. PHEO may develop as early as 8 yo in MEN2 [64], although the majority of the reported cases were >12 yo [31–34, 62].

NF1 (MIM #162200) is a multisystem autosomal dominant disorder, characterized by a progressive development (since birth) of *café au lait* spots (~100.0%), axillary/inguinal freckling (90.0%), neurofibromas (84.0%), Lisch nodules of the iris (>70.0%), typical osseous lesions (14.0%; scoliosis, sphenoid wing, and/or long bone dysplasia), and optic glioma (4.0%) [65]. NF1 also predisposes to breast, lung, and colorectal carcinomas (16.0%); PHEO (7.7%); sarcomas (7.0%); gastrointestinal stromal tumors (7.0%); melanoma (0.1–5.4%); and pancreatic neuroendocrine tumors [66–69]. The mean age at a first tumor diagnosis is 44 years [67], including PHEO (43 yo) [8]. Pediatric PHEO is associated with NF1 in 3.0% of cases, and the clinical diagnosis is often straightforward since 97.0–100.0% of patients develop at least two cardinal features of the syndrome by 8 yo [31, 65]. The youngest age of diagnosis of PHEO in NF1 is 7 years [70], but it usually develops ≥14 yo [8].

The syndrome of PHEO/PGL and somatostatinoma associated with polycythemia, caused by *HIF2A* mutations, is a new PHEO-associated cancer syndrome described initially in 2012 [71]. Patients are prone to a set of clinical features, occurring isolated or in different combinations: polycythemia since early childhood, PHEO, PGL, duodenal somatostatinomas, and retinopathy [50, 72–82]. The full syndrome—“Pacak-Zhuang” syndrome—is considered if the patient develops polycythemia, PHEO/PGL, and somatostatinoma [50]. Overall clinical manifestations and their frequency in the 62 published cases with *HIF2A* mutations are as follows: isolated polycythemia in 29 patients (45.0%); polycythemia and PHEO/PGL in nine patients (14.5%); polycythemia, PHEO/PGL, and somatostatinoma in six patients (9.6%); isolated PHEO/PGL in 14 patients (22.6%); brain hemangiomas in three patients (4.8%, one with a concomitant PGL); and duodenal gangliocytic PGL in two patients (3.2%) [50, 72–82]. The median age of diagnosis of PHEO is 40 years (range: 13–78), whereas for PGL, it is 20 years (range: 8–78) [50, 72–82]. The prevalence of *HIF2A* mutations associated with pediatric PHEO is unknown, as no case series in this age range have been published that included the analysis of this gene. Considering all ages, the prevalence of *HIF2A* mutations is estimated to be 5.3% [74, 77, 78] in cohorts of PHEO/PGL. Of these, 25.0% and 42.8% are patients that developed PHEO and PGL, respectively, at a pediatric age [50, 72, 74, 75, 77]. These tumors are initially benign and multiple, but later on, they recur frequently, requiring repeated surgeries, and develop metastases, especially PGLs [50]. Somatostatinoma occurs only in females at the median age of diagnosis of 32 years (range: 22–59), and they are always located around the duodenal ampulla [50, 72]. Considering all cases published to date, these tumors are associated with symptomatic gallbladder disease; occur in the duodenum (100.0%) and pancreas (50.0%); carry a considerable risk of recurrence (50.0%) and malignancy (50.0%); and are diagnosed after the development of PHEO/PGL [50, 72]. The majority of *HIF2A* mutations are somatic, and thus, the family history is negative. However, some patients have somatic mosaicism, where the mutation is found in tumor cells and in a fraction of normal tissues (e.g., leukocytes and buccal cells) [50, 72, 74, 78, 83]. Thus, there may be a possibility of transmission of a *HIF2A* mutation to the next generation by an affected member who has mosaicism that includes the gametes; however, such cases have never been described until now [72]. Additionally, there are seven familial cases of *HIF2A* mutations, but the majority had only polycythemia [50], and two nonrelated cases of germline mutations in adult patients with isolated PHEO [78]. This evidence has led experts to develop recommendations regarding the genetic testing and counseling, as well as to the clinical follow-up of patients with *HIF2A* mutations [50, 72].

Germline mutations in *PHD1*/*2* were reported in patients with polycythemia and PHEO/PGL [17, 53]. In this syndrome, patients develop polycythemia at a later age relative to *HIF2A* mutation carriers, but they appear to have a similar high risk of recurrent chromaffin cell tumors, especially PGL [17, 53]. To date, only a *PHD1* mutation was reported in association with pediatric PHEO. The patient was a female with no family history that presented with polycythemia diagnosed at 6 yo and developed a PHEO at 14 yo. Subsequently, she had a contralateral PHEO and a thoracic PGL [17].

### 3.3. Multifocal Disease

Pediatric patients with PHEO and PGL usually have germline mutations in the *SDHB*, *SDHD*, or *VHL* [31–33]. *SDHB* mutations cause PGL4 (MIM #115310) [13], an autosomal dominant disorder characterized mainly by the development of sympathetic abdominal (67.0%) and thoracic PGL (17.6%), parasympathetic head and neck (HN) PGL (27.5%), and/or PHEO (11.4%) [49, 84]. The mean age of presentation is 34 years, and the penetrance reaches 65.0% by 40 yo [49]. Pediatric patients with PHEO harbor an *SDHB* mutation in 13.6% of cases, and the majority develop this tumor at ≥8 yo [31]. PGL4 is associated with the highest incidence of PHEO (96.0%) of all the PGL syndromes in this age range; an *SDHB* mutation is most likely present when a pediatric PHEO occurs concomitantly with an abdominal PGL (68.0%) [28, 31, 32] but is less likely than other *SDHx* mutations when it occurs in association with thoracic (8.0%) and HN PGL (4.0%) [31]. *SDHB* mutations are also associated with the development of RCC (14%), gastrointestinal stromal tumors [GIST; 2%; isolated or associated with PGL (Carney dyad or Carney-Stratakis syndrome) or PGL and chondroma (Carney triad)] and pituitary adenomas (rare) [85–90]. Germline *SDHD* mutations predispose carriers to PGL1 (MIM #168000) [9]. This syndrome is characterized by parasympathetic HN PGL (89.0%), sympathetic thoracic PGL (16.0%), and/or PHEO (10.5%), with a particularly high incidence of multiple tumors (66.9%). The mean age of presentation is 28 years, and the penetrance reaches >80.0% by 40 yo [49, 84]. Pediatric PHEO is associated with *SDHD* mutations in 6.7% of cases. The mean age of diagnosis of PGL1 is 14 years, and the earliest presentation reported is at 5 yo [3, 31]. An *SDHD*-related PHEO is more likely—rather than *SDHB* or *VHL*—when thoracic (24.0%) or HN PGL (6.0%) is also diagnosed in pediatric patients. PGL1 is also characterized by recurrent PGL that occurs with a lower latency period in this age range [31]. Nonchromaffin cell tumors may also occur in patients with *SDHD* mutations (RCC, 8%; GIST rare, isolated, or part of Carney dyad/Carney-Stratakis syndrome/or triad; and pituitary adenomas, rare) [85–90]. PGL1 almost always manifests when the *SDHD* mutation is paternally inherited, due to a selective somatic loss of the maternal chromosome 11. Lack of the paternal chromosome 11 does not lead to tumor initiation due to a maternal oncosuppressor locus in the 11p15 region (imprinted in the father) [91]. Thus, the family history may show a “skip-generation” pattern [49, 92, 93]. Very rarely, loss of the paternal 11q (where *SDHD* allele is located) and a mitotic recombination of the maternal 11q (carrying an *SDHD* mutation) with the paternal 11p15 imprinted oncosuppressor region may lead to the phenotypic expression of the disease, inherited from the mother [49, 91–93].

VHL disease is rarely associated with PGL across the age spectrum [7, 56], but these tumors tend to occur with a higher frequency in pediatric (4.6–5.6%) compared with adult (0.96%) patients [94]. Indeed, recent case series reveal that pediatric PHEO harboring *VHL* mutations can occur in association with abdominal (20.0%) and/or thoracic (3.0%) PGL [31], with a small study reporting this phenotype in 38.0% of cases [32]. Additionally, pediatric patients with PHEO-associated *VHL* mutations have a significantly higher likelihood of new contralateral adrenal and extraadrenal tumors than have mutations in other genes [31].

*HIF2A* mutations are typically associated with the development of multifocal PGL. The youngest age reported to date is 8 years [50], and although the age of diagnosis of PGL is younger than that of PHEO, PGL may occur simultaneously (33.3%) or develop after PHEO (33.3%) in patients with *HIF2A* mutations [45, 50, 72, 73]. Additionally, PGL has a particularly high recurrence rate of new tumors in these patients during the follow-up, mainly in the abdomen [45, 50, 73]. Thus, recommendations include screening by imaging studies (MRI for pediatric patients) starting at 8 yo, and from there every 1-2 years [50]. As previously stated, *PHD1* mutations may cause a phenotype (pediatric penetrance, multifocal, and recurrent PGL) [17] similar to that of *HIF2A* mutations, as proteins coded by both genes are partners in the PHD/HIF-*α*/VHL pathway [42]. Patients with *PHD1*/*PHD2*/*HIF2A* mutations should be closely followed up with functional imaging techniques for recurrence, with ^18^F-fluorodihydroxyphenylalanine (^18^F-FDOPA) position emission tomography (PET)/computed tomography (CT) being the most accurate among all the available techniques [50]. This evidence highlights the importance of genetic screening for the delivery of the best clinical practice to patients with PHEO/PGL.

*FH* mutations are among the rare genetic etiologies of PHEO, with an estimated prevalence of 1.05% in two cohorts of PHEO/PGL patients (totalizing 670 cases) [16, 51]. Considering the seven unrelated cases published to date, the median age of diagnosis is 41 years (range: 6–70) [16, 51], with one single pediatric patient reported (14.2%) who developed a unilateral PHEO at 6 yo [51]. *FH* mutations have shown to predispose patients to PHEO and multifocal PGL (mainly abdominal) with a significantly higher rate than mutations in other PHEO susceptibility genes [16].

The familial PHEO syndrome caused by *MAX* mutations has a prevalence of <2.0% among the genetic etiologies of PHEO [14, 24]. Besides a paternal pattern of heritability (“skip-generation” pattern) [14], probands do not have a positive family history in >65.0% of cases, which may hinder the identification of a hereditary disease. The mean age of diagnosis is 32 years, and the youngest age published is 13 years [14, 24]. *MAX* mutations may predispose patients with PHEO to thoracic and abdominal PGL (18.5%) [14], including at a pediatric age (14.3%) [14, 32, 33].

### 3.4. Bilateral PHEO

Bilateral PHEO (bPHEO) at a pediatric age is associated mainly with VHL disease, and less frequently with MEN2 syndrome, PGL1, and familial PHEO caused by *MAX* mutations [14, 31–33]. VHL disease may be associated with bPHEO at presentation in 6.2% of patients, but at a pediatric age, bPHEO tends to occur with a higher frequency, ranging from 19.0% to 39.0% [31, 33, 94]. Additionally, 35.0% of pediatric patients with VHL disease and a unilateral PHEO may develop a contralateral tumor in the long-term follow-up [31].

MEN2 is associated with the highest incidence (50.0–78.0%) of bPHEO among the susceptibility genes for PHEO [95, 96], and a contralateral tumor often (16.0–40.0%) develops 1–14 years after unilateral presentations (considering all ages) [61, 95, 96]. As stated above, *RET* mutations are rare in cohorts of pediatric PHEO, but when present, there is a usually high incidence of bPHEO (66.0–100.0%) [31–36, 95]. The high frequency of high risk *RET* mutations [in particular, the NM_020975.4(RET):c.1900T>C (p.Cys634Arg)] for bPHEO in published cohorts could be an explanation, although sample bias (small sample sizes) should also be considered [32, 34, 63, 96].

PGL1 is rarely associated with bPHEO across the age spectrum [84]. However, pediatric patients with bPHEO may harbor an *SDHD* mutation in 6.9–12.5% of cases, and its presence might thus be considered in these cases [31, 33].

*MAX* mutations predispose carriers to a high risk of bPHEO or multifocal synchronous unilateral tumors (68.4%) [14, 24], including at a pediatric age (41.0%) [14].

### 3.5. Metastatic PHEO

Metastatic PHEO is defined by the presence of metastasis in tissues where chromaffin cells are not normally present (e.g., bone and lymph node) [1]. Its prevalence is reported to be 10.0%, considering all age groups [5, 21, 24]. The current rate of metastatic pediatric PHEO is difficult to establish, as most cohort studies do not distinguish between PHEO and PGL when reporting metastatic frequencies [2, 31, 32, 36]. However, two studies totalizing 95 pediatric patients reported an incidence of metastatic lesions of 8.1–12.0% [33, 35]. The majority of malignant pediatric PHEO cases are associated with PGL4, and less frequently with VHL disease, NF1, PGL5, familial PHEO due to *MAX* mutations, and *FH* mutations [14, 16, 25, 31, 32].

PGL4 is associated with the development of metastatic PHEO and/or PGL in 37.0% of patients across the age spectrum [13, 49]. Thoracic and abdominal sympathetic PGL carries the highest risk of metastasis, mainly to the lymph nodes, liver, lungs, and bones [13]. At a pediatric age, metastatic PHEO occurs in the context of *SDHB* mutations in 57.0% of cases [25]. Additionally, considering the largest case series of pediatric PHEO, the metastatic rate of PHEO and/or PGL associated with *SDHB* mutations (18.2–26.0%) was significantly higher when compared with that of carriers of mutations in other susceptibility genes; a lower lifetime expectancy of carriers of *SHDB* mutations was also recognized [31]. Thus, these patients need a rigorous follow-up for timely detection of metastatic disease. When comparing all the available functional imaging techniques for this purpose, the most accurate for patients with *SDHx* mutations is [^68^Ga]-DOTA(0)-Tyr(3)-octreotate ([^68^Ga]-DOTATATE) PET/CT, followed by [^18^F]-fluoro-2-deoxy-d-glucose PET/CT [97]. Again, this evidence emphasizes the importance of knowing the patient genotype for the delivery of precision medicine.

VHL disease has a prevalence of metastatic PHEO that is variable between studies, ranging from 0.0% to 8.0% [6, 98, 99], with an extensive review quoting it at 3.4% [24]. In pediatric patients, the reported rate of metastatic tumors was estimated to be 12.5–28.0% in two small sample studies [25, 32], but in the largest cohort, the incidence of malignant PHEO and/or PGL was 4.3% [31]. While the metastatic risk does not appear to be high, it may be worth to consider the analysis of *VHL* in a pediatric patient with a metastatic PHEO, due to the high prevalence of *VHL* mutations at this age range [31–33].

Although associated with PHEO in ≤6.0% of cases, NF1 has a prevalence of metastatic PHEO of 7.7–12.0% [8, 100]. In pediatric patients with NF1, metastatic PHEO occurs at a rate of 33.3–66.6% [31, 100], and similar to PGL4, these individuals have lower lifetime expectancy [31]. However, NF1 patients are represented in small subsamples (three to six patients) of pediatric PHEO cohort studies [31, 100], precluding the establishment of their metastatic risk.

The familial PHEO syndrome caused by *MAX* mutations is associated with metastatic PHEO in 10.5% of cases, considering all ages [14]. No cases of *MAX*-related metastatic tumors have been reported so far in cohorts of pediatric PHEO [31, 32]. However, in a study of patients with PHEO-associated *MAX* mutations, five patients were ≤18 yo, of which one had a metastatic tumor [14].

PGL5 (MIM #614165) is caused by mutations in the subunit A of the SDH complex [12], which are found in 3.0% of all PHEO/PGL patients [101, 102]. This syndrome has a median age of presentation of 33 years, and the penetrance reaches 38% by 40 yo [12, 28, 31, 101]. The youngest age reported is 8 years, with four pediatric cases published to date [31, 33, 101]. *SDHA* mutations predispose patients to HN PGL (38.9%), abdominal PGL (27.8%), and unilateral PHEO (24.0%) [12, 28, 31, 33, 101–107]. *SDHA* mutations also confer susceptibility to GIST (30% of SDHx deficient GIST) and pituitary adenomas (rare) [85–100, 108]. The metastatic rate of pediatric PHEO in PGL5 is difficult to establish, due to its rarity. In the largest case series (totalizing 38 patients) of PHEO/PGL-associated *SDHA* mutations, the reported prevalence of metastatic PHEO/PGL in general was 11% [101]. Of the four pediatric cases, three presented with unilateral PHEO, one of which was metastatic, and one displayed an abdominal PGL [31, 33, 101].

*FH* mutations are associated with a high rate of metastatic PHEO. Although only 7 PHEO/PGL cases have been reported to date, three have developed metastasis, of which two (28.6%) were PHEO [16]. In the largest collaborative cohort study of PHEO/PGL where the main susceptibility genes were analyzed, PHEO/PGL caused by *FH* mutations had a significantly higher rate of malignancy than had tumors associated with other gene mutations [16]. Thus, similar to *NF1* and *MAX* mutations, where small samples have been shown a propensity for malignant PHEO [8, 14], it may be important to maintain a higher index of suspicion for the presence of malignancy when following up pediatric patients with PHEO-associated *SDHA* and *FH* mutations.

### 3.6. Solitary PHEO

A unilateral PHEO is the most common presentation of this tumor in clinical grounds [2, 5, 31]. In pediatric patients with a solitary PHEO and an associated gene mutation, *VHL* accounts for the vast majority of cases, followed by *SDHB* and *SDHD* [3, 31–34]. Considering 84 patients with a solitary and apparently nonsyndromic PHEO (the largest case series at a pediatric age), mutations in *VHL*, *SDHB*, and *SDHD* were found in 62, 8, and 3 cases, respectively [31–33].

### 3.7. Ancillary Surveys to Support the Genetic Screening

The pattern of catecholamine secreted by the PHEO may yield clues to the genetic background of the patient, especially when no family history or syndromic features are evident [23, 27, 109]. PHEO associated with MEN2 or NF1 usually produces and/or cosecretes norepinephrine/normetanephrine and epinephrine/metanephrine [27]. However, PHEO associated with VHL disease produces and/or secretes norepinephrine/normetanephrine, but not epinephrine/metanephrine, due to the lack of the enzyme that catalyzes the conversion of norepinephrine to epinephrine [27]. Similar to VHL disease, tumors associated with *SDHx*, *HIF2A*, and *FH* mutations produce and/or secrete noradrenaline/normetanephrine but rarely adrenaline/metanephrine [16, 27, 50]. However, PHEO associated with *SDHx* mutations also produce and/or secrete dopamine/methoxytyramine, which is rarely detected in VHL disease [27]. In agreement with these findings, the discriminatory rate of the pattern of catecholamine production and/or secretion between NF1/RET- (normetanephrine and metanephrine) and *VHL*/*SDHx*-associated PHEO (normetanephrine but not metanephrine) was quoted at 99.0%. This last cluster may be correctly discriminated in 78.0% of cases by the levels of methoxytyramine (elevated in *SDHx*, but not in *VHL*-associated PHEO) [27]. *MAX*-associated PHEO secretes high levels of normetanephrine and moderate levels of metanephrine [14].

Immunohistochemistry to SDHA and SDHB in the tumor sample may also provide useful information to prioritize the genetic screenings [28, 102, 110]. Lack of staining for SDHB in PHEO is highly suggestive of germline mutations in *SDHx* genes (90.0%), whereas immunonegative staining for SDHA (75.0%) is indicative of *SDHA* mutations [28]. False negatives (positive or weakly positive staining) may occur in *SDHD*-related lesions for SDHB staining [111], and SDHD immunohistochemistry may aid in these cases (positive staining predicts *SDHx* mutations) [112]. Contrarily, all *RET-*, *HIF2A-*, and *MAX-* and majority of *NF1*- (95.0%) and *VHL*-associated PHEO (84.0%) show positive immunostaining for subunits A/B of the SDH complex [28, 110].

Functional imaging is used in the management of PHEO to localize the primary tumor or to define the tumor burden of a metastatic PHEO that may be missed by CT or MRI, and to characterize the metabolic activity of PHEO/PGL for therapeutic purposes (e.g., pretreatment uptake avidity evaluation of metastatic disease with ^123^I-metaiodobenzylguanidine scintigraphy) [23, 113]. The ^18^F-FDOPA PET/CT is a highly accurate functional imaging tool in the investigation of PHEO [30]. However, false-negative results may infrequently occur, mainly with abdominal PGL or PHEO. These missed lesions on ^18^F-FDOPA PET/CT are often associated with *SDHB* and *SDHD* mutations, and it is worth to consider focused genetic screening for *SDHx* mutations in ^18^F-FDOPA PET/CT-negative PHEO/PGL [30, 113].

## 4. Next-Generation Sequencing: A New Pediatric PHEO Diagnostic Tool?

Targeted next-generation sequencing (NGS) is a new technology that processes DNA samples for simultaneous parallel sequencing of multiple genes [114, 116]. Considering the high number of PHEO-related genes, NGS is attractive in this context. Indeed, the application of NGS in cohorts of patients with PHEO/PGL has proved to be faster with lower costs than has that of the conventional Sanger sequencing techniques [116, 117]. However, some limitations of NGS may need to be resolved before its full implementation in the everyday practice, namely, the clinical relevance of variants of uncertain significance or methodological errors induced by repetitive DNA sequences and pseudogenes [113–115]. Additionally, specific NGS panels may need to be constructed for samples of pediatric patients with PHEO, as several susceptibility genes analyzed in the current commercially available NGS technologies have never been reported in this age range (e.g., *TMEM127* and *SDHAF2*).

## 5. Conclusions

Genetic testing is of paramount importance in pediatric patients with an apparently sporadic PHEO, because (1) the rate of mutations found in this clinical setting is close to 80.0%; (2) 10 PHEO-associated genes have been reported in pediatric patients, each gene conferring distinct profiles of propensity for the development of chromaffin and nonchromaffin cell tumors and for biological behaviors; and (3) it allows for tailoring specific diagnostic, treatment, and surveillance programs to these patients, taking into account the germline mutation founded and its genotype-phenotype correlation [5, 22, 23, 30–33, 50, 118].

**Figure 1:**
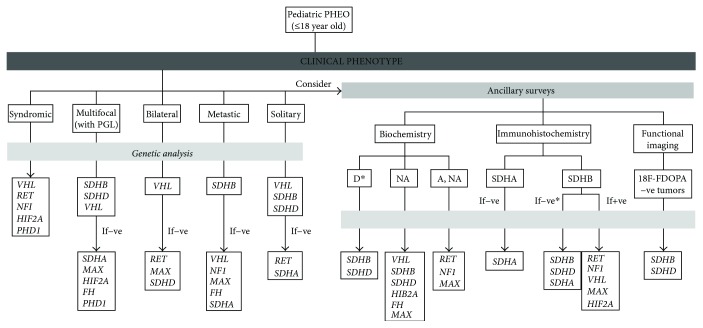
Algorithm for genetic testing in patients with pediatric PHEO, according to clinical presentation, tumor immunohistochemistry, and biochemical profiles. −ve: negative; +ve: positive; ^18^F-FDOPA: ^18^F-fluorodihydroxyphenylalanine; PHEO: pheochromocytoma; PGL: paraganglioma; A: adrenaline; D: dopamine; NA: noradrenaline; *FH*: fumarate hydratase gene; *HIF2A*: hypoxia-inducible factor 2 alpha gene; *MAX*: Myc-associated protein X gene; *NF1*: neurofibromatosis type 1 gene; *PHD1*: prolyl hydroxylase domain protein 1 gene; *RET*: rearranged during transfection gene; *SDHA*: succinate dehydrogenase subunit A gene; SDHA: succinate dehydrogenase subunit A protein; *SDHB*: succinate dehydrogenase subunit B gene; SDHB: succinate dehydrogenase subunit B protein; *SDHC*: succinate dehydrogenase subunit C gene; *SDHD*: succinate dehydrogenase subunit D gene; *VHL*: von Hippel-Lindau gene. ^∗^Consider *SDHC* mutations (PGL; PHEO reported only in patients > 18 years).

Considering the high costs of genetic screenings and the increasing number of susceptibility genes for PHEO, clinicians should follow a phenotype-driven algorithm when requesting a genetic test (see Figure 1 and Table 1) [22, 23]. Several genes (e.g., *TMEM127* and *PHD2*) have only been reported in adults [10, 11, 15, 17–20, 31–33, 52, 53, 101], and the genetic analysis of a pediatric PHEO should initially disregard them. *VHL*, *SDHB*, and *SDHD* are the most frequently mutated, whereas other genes are rarely found in pediatric patients [23, 31–33]. The high rate of malignancy with *SDHB* mutations demands extensive initial diagnostic surveys and a close surveillance program [25, 118]. Similarly, the greater likelihood of recurrent tumors in pediatric patients with *VHL* and *SDHD* mutations needs a proactive long-term follow-up [31]. Also, pediatric carriers of PHEO-associated mutations may differ in the clinical phenotype when compared to adult carriers (e.g., the higher rate of bPHEO and PGL in pediatric VHL disease) [31], findings that may change the clinical attitude regarding the extent of diagnostic and follow-up strategies. Due to the rarity of PHEO-associated mutations in other susceptibility genes, data retrieved from the published literature may hinder the establishment of genotype-phenotype correlations for some of these genes. Nevertheless, new mutations have been described at a pediatric age that correlate with specific phenotypes (e.g., *HIF2A* mutations and “Pacak-Zhuang” syndrome), opening new options for sequential genetic testing approach and for individualized strategies regarding diagnosis, treatment, long-term follow-up, and genetic counseling [50, 51].

## Figures and Tables

**Table 1:** Clinical features of PHEO-associated genes at a pediatric age.

Clinical features						Ancillary surveys	
PHEO-associated genes	Youngest age at diagnosis (years)	Most common associated tumors and features	Frequency (%)				
			Multifocal tumors	Bilateral PHEO	Metastatic PHEO	Biochemical phenotype	SDHA/B IHC
*VHL*	4	(i) CNS hemangiomas	4.6–5.6	19.0–39.0	4.3	NA	+ve/+ve
		(ii) Renal cysts					
		(iii) RCC					
		(iv) pNET					
		(v) Pancreatic cysts					
		(vi) Abdominal PGL					
		(vi) Thoracic PGL					

*RET*	8	(i) MEN2A (MTC, pHPT)	Rare	66.0–100.0	Rare	A	+ve/+ve
		(ii) MEN2B (MTC, marfanoid habitus, ganglioneuromatosis of the gut/oral mucosa)					

*NF1*	7	(i) *Café au lait* spots	Rare	Rare	33.3–66.6^∗^	A	+ve/+ve
		(ii) Axillary/inguinal freckling					
		(iii) Neurofibromas					
		(iv) Lisch nodules of the iris					
		(v) Typical osseous lesions					
		(vi) Optic glioma					
		(vii) Carcinomas (breast, lung, colorectal)					
		(viii) Sarcomas, GIST					
		(ix) Melanoma					

*SDHB*	6	(i) Abdominal PGL	68.0	Rare	57.0	NA; D	+ve/−ve
		(ii) Thoracic PGL					
		(iii) HN PGL					
		(iv) RCC					
		(v) GIST					
		(vi) Pituitary adenoma					
		(vii) Chondroma					

*SDHD*	5	(i) HN PGL	66.9	6.9–12.5	Rare	NA; D	+ve/−ve
		(ii) Thoracic PGL					
		(iii) RCC					
		(iv) GIST					
		(v) Pituitary adenoma					
		(vi) Chondroma					

*SDHA*	8	(i) HN PGL	9.0	4.0	11.0	NA; D	−ve/−ve
		(ii) Abdominal PGL					
		(iii) GIST					
		(iv) Pituitary adenomas					

*HIF2A*	8	(i) Polycythemia since early childhood	66.6	Rare	Rare^∗∗^	NA	+ve/+ve
		(ii) Abdominal PGL					
		(iii) Duodenal somatostatinomas					
		(iv) Retinopathy					

*PHD1*	14	(i) Polycythemia	100	ND	ND	NA	ND
		(ii) Abdominal/thoracic PGL					

*MAX*	13	(i) Abdominal PGL	14.3	41.0	20.0	NA; NA, A	+ve/+ve
		(ii) Thoracic PGL					

*FH*	6	(i) Abdominal PGL	42.8	0.0	28.6	NA	ND
		(ii) Thoracic PGL					

−ve: negative; +ve: positive; CNS: central nervous system; GIST: gastrointestinal stromal tumor; HN: head and neck; MTC: medullary thyroid carcinoma; PHEO: pheochromocytoma; PGL: paraganglioma; RCC: renal cell carcinoma; pNET: pancreatic neuroendocrine tumor; A: adrenaline; D: dopamine; IHC: immunohistochemistry; NA: noradrenaline; *FH*: fumarate hydratase gene; *HIF2A*: hypoxia-inducible factor 2 alpha gene; *MAX*: Myc-associated protein X gene; MEN2A: multiple endocrine neoplasia type 2A; MEN2B: multiple endocrine neoplasia type 2B; *NF1*: neurofibromatosis type 1 gene; *PHD1*: prolyl hydroxylase type 1 gene; *RET*: rearranged during transfection gene; *SDHA*: succinate dehydrogenase subunit A gene; SDHA: succinate dehydrogenase subunit A protein; *SDHB*: succinate dehydrogenase subunit B gene; SDHB: succinate dehydrogenase subunit B protein; *SDH*C: succinate dehydrogenase subunit C gene; *SDHD*: succinate dehydrogenase subunit D gene; *VHL*: von Hippel-Lindau gene; ND: not defined. ^∗^Small samples in case series. ^∗∗^Metastatic PGL: 29%.

## Abbreviations

ADM: Adrenomedullin
ATRX: X-Linked alpha thalassemia mental retardation
bPHEO: Bilateral pheochromocytoma
BNIP3: BCL2/adenovirus E1B 19 kDa protein-interacting protein 3
CT: Computed tomography
CCND1: G1/S-Specific cyclin-D1
CSDE1: Cold shock domain-containing E1
EPO: Erythropoietin gene
^18^F-FDOPA: ^18^F-Fluorodihydroxyphenylalanine
FH: Fumarate hydratase
[^68^Ga]-DOTATATE: [^68^Ga]-DOTA(0)-Tyr(3)-octreotate
GIST: Gastrointestinal stromal tumors
HRAS: Harvey rat sarcoma viral oncogene homolog
HK1: Hexokinase 1
HK2: Hexokinase 2
HIF-*α*: Hypoxia-inducible factor alpha
HIF2A: Hypoxia-inducible factor 2 alpha
KIF1B*β*: Kinesin family member 1B
MAML3: Mastermind-like transcriptional coactivator 3
MTC: Medullary thyroid carcinoma
MEN2: Multiple endocrine neoplasia type 2
MAX: Myc-Associated protein X
NF1: Neurofibromatosis type 1
NGS: Next-generation sequencing
PGL: Paraganglioma
PGL1: Paraganglioma syndrome type 1
PGL2: Paraganglioma syndrome type 2
PGL3: Paraganglioma syndrome type 3
PGL4: Paraganglioma syndrome type 4
PGL5: Paraganglioma syndrome type 5
PHEO: Pheochromocytoma
PET: Position emission tomography
PHD1: Prolyl hydroxylase type 1
PHD2: Prolyl hydroxylase type 2
RET: Rearranged during transfection
RCC: Renal cell carcinomas
SDH: Succinate dehydrogenase
SDHA: Succinate dehydrogenase subunit A
SDHB: Succinate dehydrogenase subunit B
SDHC: Succinate dehydrogenase subunit C
SDHD: Succinate dehydrogenase subunit D
SDHAF2: Succinate dehydrogenase assembly factor 2
TGFB: Transforming growth factor beta gene
TMEM127: Transmembrane protein 127
VEGFA: Vascular endothelial growth factor A
VHL: Von Hippel-Lindau
Yo: Years old.
